# Privatizing the Welfare State

**DOI:** 10.1093/geroni/igaa057.2540

**Published:** 2020-12-16

**Authors:** Pamela Herd

**Affiliations:** Georgetown University, Washington, District of Columbia, United States

## Abstract

The growth of the private sector in the Medicare and Medicaid programs is a sea change, leading many to argue that the old age welfare state is effectively becoming privatized. I examine these trends, but focus on the consequences for how older adults experience their interactions with government. In particular, I examine how privatization increases administrative burden for beneficiaries. Older adults must navigate hundreds of choices, leading to significant confusion. Most fail to pick policies that maximize their benefits and reduce their cost. This confusion harms beneficiaries. They end up with suboptimal coverage, with increased out of pocket costs and decreased access to care. The confusion, however, generates profits for insurers. Part of a symposium sponsored by the Women's Issues Interest Group.

